# Supplementary figures and images for: A novel replication initiation region encoded in a widespread Acinetobacter plasmid lineage carrying a blaNDM-1 gene
Source: PLoS One. 2024 May 31;19(5):e0303976. doi: 10.1371/journal.pone.0303976 (PMC11142715; doi:10.1371/journal.pone.0303976)

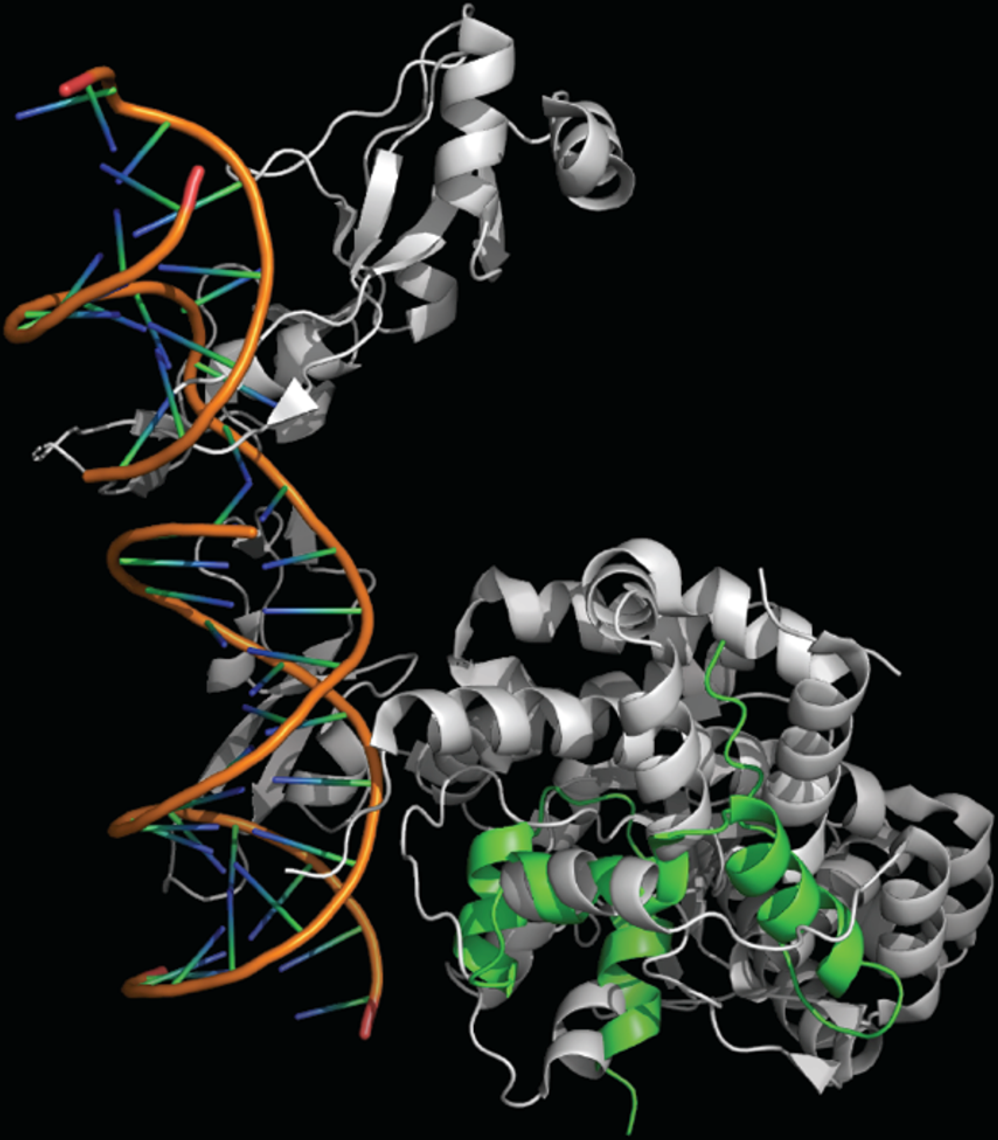

Supplement: S1 Fig — (TIF) [file pone.0303976.s001.tif]

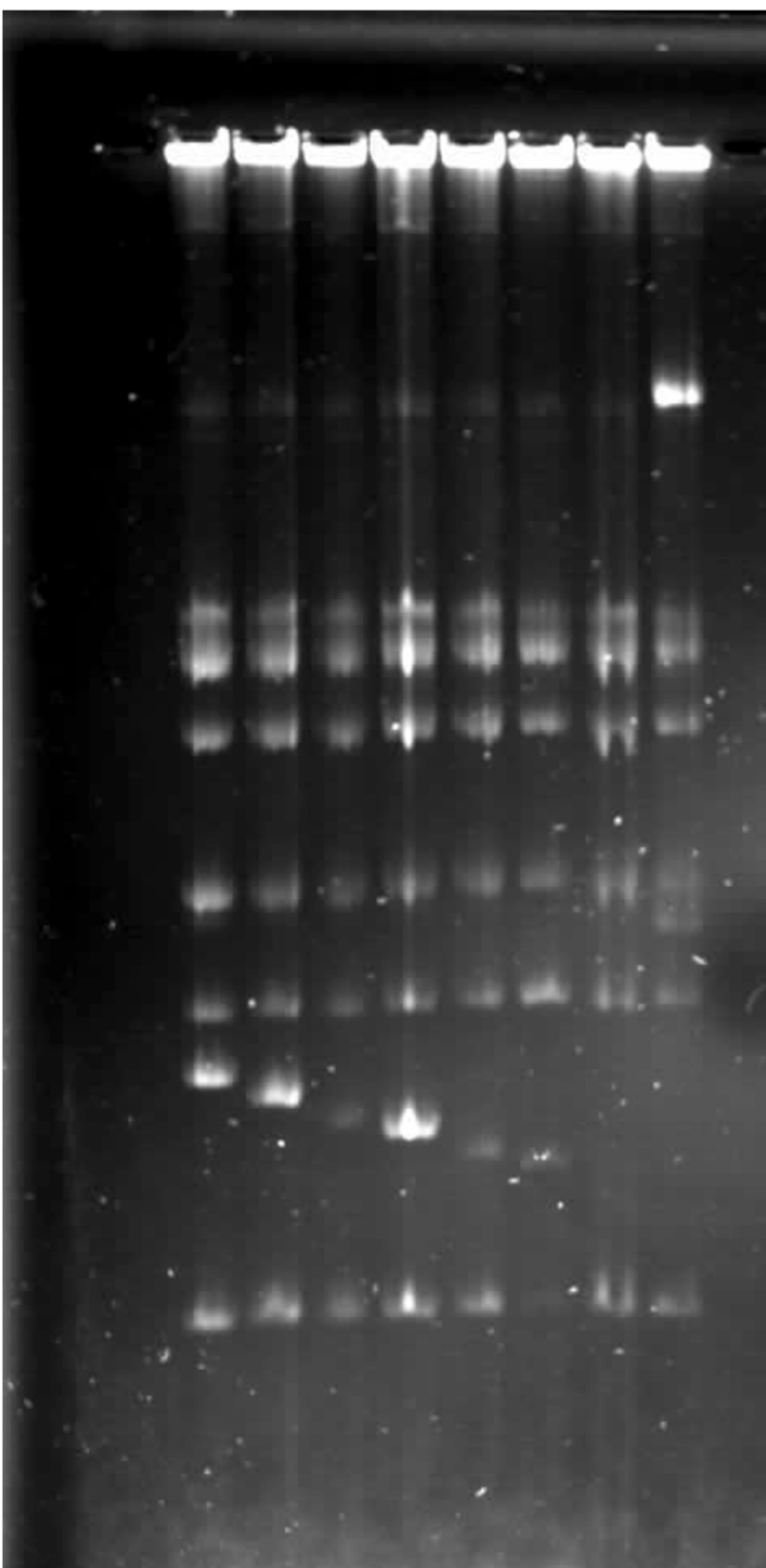

Supplement: S1 File — (PDF) [file pone.0303976.s002.pdf]
