# Supplementary material for: A novel replication initiation region encoded in a widespread Acinetobacter plasmid lineage carrying a blaNDM-1 gene
Source: PLoS One. 2024 May 31;19(5):e0303976. doi: 10.1371/journal.pone.0303976 (PMC11142715; doi:10.1371/journal.pone.0303976)
Supplement: S1 Table — (PDF) [file pone.0303976.s003.pdf]

Name	Sequence	Restriction site
pAhaeAN54e_1F	**CTCGAG**CCTAAAGGGGGTGTAAACGAAGTGTC	XhoI
pAhaeAN54e_2R	**CTCGAG**ATTATTCATAAAACCGCCCTCATTTAATATTTAGGT	XhoI
pAhaeAN54e_3F	**CTCGAG**ACCTAAATATTAAATGAGGGCGGTTTTATGAATAAT	XhoI
pAhaeAN54e_4R	**CTCGAG**GCTTAGTACGCTTAAAAATAATTTTCTTC	XhoI
pAhaeAN54e_5F	AGTC**CTCGAG**AGTCTAAGTTTCTTTTAATGATGATTTTG	XhoI
pAhaeAN54e_6R	**CTCGAG**GATCTGCAGGCTTAGATACTGAAATCTGAAGTGAG	XhoI
pAhaeAN54e_7F	**CTCGAG**TATGTATGTTCTATCAGGAAGATTGTAGCG	XhoI
pAhaeAN54e_8R	**CTCGAGG**TTCAGATGGTATGAATCAATTGCTAG	XhoI
F5Xho-Up	GATA**CTCGAG**ATCATGTTCATTGATTGGGCTTAGATAAGG	XhoI
F5Pst-Low	GAT**CTGCAG**GCATTCAAAGTCTGGCTCAATTAATC	PstI
pAhaeAN54e_11F	**CTCGAG**TTTCAGATACGAACGTAAATTACCCATACC	XhoI
pAhaeAN54e_12R	**CTCGAG**GTTGCAACTTTATTATTCAAGAGGTGGACA	XhoI
pAhaeAN54e_13F	**CTCGAG**TATCACACCCTGAATTTCTACAGCC	XhoI
pAhaeAN54e_14R	**CTCGAG**TTGTACTCACAAAACAATCAAGGGG	XhoI
pAhaeAN54e_15F	**CTCGAG**TAGCCTTATCGGTTATAGAGTTATAACAG	XhoI
pAhaeAN54e_16R	**CTCGAG**TCAGTGTTATTGCTCATGTTCTTTTCC	XhoI
F9Xho-Up	ATA**CTCGAG**CAATCAATGAAATTAAGGGCTATCGTG	XhoI
F9PstI-Low	GAT**CTGCAG**GGACTTTCAAGATAGGCTCTTGAG	PstI
F10XhoI-Up	GATA**CTCGAG**GATTTAAGTGGGTTATGAGTGCGG	XhoI
F10PstI-Low	GAT**CTGCAG**GATTGCATCATTGTTATTTTGGTCG	PstI
pAhaeAN54e_21F	**CTCGAG**AGCGTATTGCAGAATTGCAAACAC	XhoI
pAhaeAN54e_22R	**CTCGAG**TACGCCAACGAAACAGCAAATTAG	XhoI
F8-B-up	CAG**CTCGAG**GTAAAGCTTTGAGCAATCAAC	XhoI
F8-C-Low2	CAG**GAGCTC**TCAGTGTTATTGCTCATGTTC	SacI
BCc-1-Up	CA**GGGTACC**CTATGATATTTCAATAAATAGC	KpnI
BCc-1.2-Up	CA**GGGTACC**CTATATTCTTCGGTACACAGT	KpnI
B-Low-215	CAG**GAGCTC**TTGTTATTTAAAGTCTTTATAAGT	SacI
B-Low-107	CAG**GAGCTC**CTTAGTCGTTTTCGCTTC	SacI
B-Low-70	CAG**GAGCTC**TTGGTGAGATTGAGCAGA	SacI
F8-AB-Low	CAG**GAGCTC**ATATTAATTTGTACTCACAAAACA	SacI
FShR1-up	CTAAGCAAAAAAAATTGCTTA**AAGCTT**CTATCTTGAATATTGCCG	HindIII
FShR1-low	CGGCAATATTCAAGATAG**AAGCTT**TAAGCAATTTTTTTTGCTTAG	HindIII
FShR2-Up	CTTATTTATGCCAGTTCCTAGCG**AAGCTT**CTGCGTCCCCGCCTATTGTG	HindIII
FShR2-Low	ACAATAGGCGGGGACGCAG**AAGCTT**CGCTAGGAACTGGCATAAATAAG	HindIII
FSh-R3-up	CAAACCTTATGATCTTCGTT**AAGCTT**TGTTTACCGCATCAAATAAC	HindIII
FSh-R3-Low	GTTATTTGATGCGGTAAACA**AAGCTT**AACGAAGATCATAAGGTTTG	HindIII

S1 Table.
