# Supplementary material for: A novel replication initiation region encoded in a widespread Acinetobacter plasmid lineage carrying a blaNDM-1 gene
Source: PLoS One. 2024 May 31;19(5):e0303976. doi: 10.1371/journal.pone.0303976 (PMC11142715; doi:10.1371/journal.pone.0303976)
Supplement: S2 Table — Plasmid Blast hits when de DNA sequence of the minimal replication region of plasmid pAhaeAN54e was used as query (100% sequence identity and 100% sequence coverage). Lenght: plasmid sizes. NDM: P (present); ND (not determined), A (absence), GIM (metallo-lactamase GIM-1). (PDF) [file pone.0303976.s004.pdf]

S2 Table 2

Description	Scientific Name	Max Score	Total Score	Query Cover	E value	Per. ident	Accession	Lenght	NDM
Acinetobacter lwoffii SU1904 plasmid pSU1904NDM DNA, complete sequence	Acinetobacter lwoffii	1541	1541	100%	0	100	LC537594.1	43651 bp	P
Acinetobacter baumannii strain AB17 plasmid pAB17, complete sequence	Acinetobacter baumannii	1541	1541	100%	0	100	MT002974.1	41087 bp	P
Acinetobacter indicus strain TQ18 plasmid p18TQ-NDM, complete sequence	Acinetobacter indicus	1541	1541	100%	0	100	CP045133.1	40439 bp	P
Acinetobacter indicus strain TQ23 plasmid p23TQ-NDM, complete sequence	Acinetobacter indicus	1541	1541	100%	0	100	CP045197.1	41393 pb	P
Acinetobacter indicus strain TQ04 plasmid p4TQ-NDM, complete sequence	Acinetobacter indicus	1541	1541	100%	0	100	CP045130.1	41086 pb	P
Acinetobacter pittii SU1805 plasmid pSU1805NDM DNA, complete sequence	Acinetobacter pittii	1541	1541	100%	0	100	LC483156.1	41022 bp	P
Acinetobacter haemolyticus strain AN54 plasmid pAhaeAN54e, complete sequence	Acinetobacter haemolyticus	1541	1541	100%	0	100	CP041229.1	45460 bp	P
Acinetobacter cumulans strain WCHAc060092 plasmid pNDM1_060092, complete sequence	Acinetobacter cumulans	1541	1541	100%	0	100	CP035935.1	48560 bp	P
Escherichia coli strain WPB121 chromosome	Escherichia coli	1541	1541	100%	0	100	CP034426.1	4637028 bp	ND
Acinetobacter baumannii strain WPB103 chromosome	Acinetobacter baumannii	1541	1541	100%	0	100	CP034427.1	3240866 bp	ND
Acinetobacter sp. WCHAc010034 plasmid pNDM1_010034, complete sequence	Acinetobacter sp. WCHAc010034	1541	1541	100%	0	100	CP032278.1	49649 bp	P
Acinetobacter chinensis strain WCHAc010005 plasmid pNDM1_010005, complete sequence	Acinetobacter chinensis	1541	1541	100%	0	100	CP032132.1	39357 bp	P
Acinetobacter nosocomialis strain IEC38057 plasmid pIEC38057, complete sequence	Acinetobacter nosocomialis	1541	1541	100%	0	100	MK053934.1	41085 bp	P
Acinetobacter baumannii strain IEC383 plasmid pIEC383	Acinetobacter baumannii	1541	1541	100%	0	100	MK053932.1	47283 bp	P
Acinetobacter baumannii strain AR_0088 plasmid unnamed2, complete sequence	Acinetobacter baumannii	1541	1541	100%	0	100	CP027532.1	41087 bp	P
Acinetobacter sp. ACNIH1 plasmid pNDM-9c17, complete sequence	Acinetobacter sp. ACNIH1	1541	1541	100%	0	100	CP026425.1	39361 bp	P
Acinetobacter baumannii strain ABNIH28 plasmid pNDM-0285, complete sequence	Acinetobacter baumannii	1541	1541	100%	0	100	CP026127.1	39359 bp	P
Providencia rettgeri strain 06-1619 plasmid p06-1619-NDM, complete sequence	Providencia rettgeri	1541	1541	100%	0	100	KX832928.1	54712 bp	P
Acinetobacter sp. SU8482 plasmid pSU8482NDM DNA, complete sequence	Acinetobacter sp.	1541	1541	100%	0	100	LC771431.1	42372 bp	P
Acinetobacter johnsonii SU8379 plasmid pSU8379NDM DNA, complete sequence	Acinetobacter johnsonii	1541	1541	100%	0	100	LC771430.1	51560 bp	P
Acinetobacter bereziniae strain UCO-554 plasmid unnamed1, complete sequence	Acinetobacter bereziniae	1541	1541	100%	0	100	CP123919.1	47274 bp	NA
Acinetobacter bereziniae strain UCO-553 plasmid unnamed1, complete sequence	Acinetobacter bereziniae	1541	1541	100%	0	100	CP123913.1	38106 bp	NA
Acinetobacter dijkshoorniae strain JVAP01 plasmid pNDM-JVAP01, complete sequence	Acinetobacter lactucae	1541	1541	100%	0	100	KM923969.1	47268 bp	P
Acinetobacter sp. JN247 plasmid pNDM-JN02, complete sequence	Acinetobacter sp. JN247	1541	1541	100%	0	100	KM210088.1	41084 bp	P
Acinetobacter lwoffii strain JN49-1 plasmid pNDM-JN01, complete sequence	Acinetobacter lwoffii	1541	1541	100%	0	100	KM210086.1	41084 bp	P
Acinetobacter nosocomialis strain 6411 plasmid p6411-9.012kb, complete sequence	Acinetobacter nosocomialis	1541	1541	100%	0	100	CP010370.2	47274 bp	P
Acinetobacter baumannii strain 6200 plasmid p6200-47.274kb, complete sequence	Acinetobacter baumannii	1541	1541	100%	0	100	CP010399.1	47274 bp	A
Acinetobacter baumannii strain OC061 plasmid p1OC061, complete sequence	Acinetobacter baumannii	1541	1541	100%	0	100	CP087301.1	47260 bp	A (GIM)
Acinetobacter pittii strain Acinetobacter pitti plasmid pNDM-AP, complete sequence	Acinetobacter pittii	1541	1541	100%	0	100	KJ003839.1	39364 bp	P
Acinetobacter lwoffii strain Iz4b plasmid pNDM-Iz4b, complete sequence	Acinetobacter lwoffii	1541	1541	100%	0	100	KJ547696.1	46570 bp	P
Acinetobacter sp. M131 plasmid pM131_NDM1, complete sequence	Acinetobacter sp. M131	1541	1541	100%	0	100	JX072963.1	47271 bp	P
Acinetobacter bereziniae strain CHI-40-1 plasmid pNDM-40-1, complete sequence	Acinetobacter bereziniae	1541	1541	100%	0	100	KF702385.1	45826 bp	P
Acinetobacter johnsonii strain MR1 plasmid pMRB, complete sequence	Acinetobacter johnsonii	1541	1541	100%	0	100	CP079750.1	41087 bp	P
Acinetobacter baumannii ZW85-1 plasmid pAbNDM-1, complete sequence	Acinetobacter baumannii ZW85-1	1541	1541	100%	0	100	JN377410.2	48368 bp	P
Acinetobacter sp. strain FL51 plasmid pNDM-FL51, complete sequence	Acinetobacter sp.	1541	1541	100%	0	100	MW073138.1	41068 bp	P
Acinetobacter baumannii strain GF216 plasmid pNDM-AB, complete sequence	Acinetobacter baumannii	1541	1541	100%	0	100	KC503911.1	47098 bp	P
Acinetobacter lwoffii strain WJ10659 plasmid pNDM-BJ02, complete sequence	Acinetobacter lwoffii	1541	1541	100%	0	100	JQ060896.1	46165 bp	P
Acinetobacter lwoffii strain WJ10621 plasmid pNDM-BJ01, complete sequence	Acinetobacter lwoffii	1541	1541	100%	0	100	JQ001791.1	47274 bp	P
Acinetobacter baumannii OCU_Ac16a plasmid pOCU_Ac16a_2 DNA, complete sequence	Acinetobacter baumannii	1541	1541	100%	0	100	AP023079.1	41087 bp	P
Acinetobacter sp. WCHAc010052 plasmid pNDM1_010052, complete sequence	Acinetobacter sp. WCHAc010052	1131	1542	100%	0	100	CP032142.1	39365 bp	P
