# Supplementary material for: A novel replication initiation region encoded in a widespread Acinetobacter plasmid lineage carrying a blaNDM-1 gene
Source: PLoS One. 2024 May 31;19(5):e0303976. doi: 10.1371/journal.pone.0303976 (PMC11142715; doi:10.1371/journal.pone.0303976)
Supplement: Attachment — Submitted filename: Response_to_Reviewers.docx [file pone.0303976.s005.docx]

**ANSWER TO REVIEWERS**

5. Review Comments to the Authors

Please use the space provided to explain your answers to the questions above. You may also include additional comments for the author, including concerns about dual publication, research ethics, or publication ethics. (Please upload your review as an attachment if it exceeds 20,000 characters)

Reviewer #1: The manuscript entitled "A novel replication initiation region encoded in a widespread Acinetobacter plasmid lineage carrying a blaNDM-1 gene" is well written and includes an interesting work therefore, I only have very minor comments:

Line 78: the sentence "always occurs near transposons, such as IS5, IS26, ISAba125, IS3000, ISCR1, Tn3, Tn125, and Tn3000". Amongst those listed IS5, IS26, ISAba125, IS3000, ISCR1 are not transposons, they are insertion sequences. Please modify the sentence.

Line 219: Change this heading to "in-silico Structural models of the peptides

**Answer: Changed as suggested.**

Line 239: Please include the GenBank accession number for pAhaeAN54e.

**Answer: Included as suggested.**

I'd replace Figure 1 with a more advanced Figure.

Reviewer #2: The article by Bello-Lopez et al presents some interesting findings around the identification of a region in some *Acinetobacter* plasmids that is required for replication. Until now these plasmids were not known to contain a replication initiation region, and so this study furthers our knowledge of the biology of this group of plasmids. The study is overall well written and has been conducted thoroughly. There are a few points that I think the authors should address:

1. I did not notice any statistical analyses of data such as differences (or not) in conjugation frequencies, rate of plasmid loss, and growth rates with different constructs. These should be carried out to determine whether any differences reach statistical significance, and these data displayed on the appropriate figures e.g. Figure 5A and B.

**Answer: statistics now included in the figure legend.**

2. I didn't understand Figure 2. From the methods section I understood that if a collection of yellow colonies grew in the center of the plate, then these represented the transconjugants. In the results (lines 264-266) it refers to figure 2 and days that only pDOG-G was able to produce transconjugants, but Figure 2 appears to show transconjugants for pDOG-G, pDOG-B215 and pDOG-B103. Please can further explanation be provided to explain this. If the authors have the pictures, it might be worth showing the plates for the other constructs that didn't produce transconjugants as well, rather than just the positive ones.

**Answer: The new figure 2 include all constructs**

3. The legend for Figure 3 refers to pDOG-BCc, but elsewhere in the manuscript there are two plasmids called pDOG-BCc1 and pDOG-BCc2. Which is being referred to in Figure 3? Further, the legend refers to a plasmid called pDOG-B123 - should this be pDOG-B103?

**Answer: corrections revised and included in the new text.**

4. In places, mainly in the introduction, the language needs to be tightened up a bit as it is sometimes inconsistent, misleading, or erroneous. Examples include:

Line 59: change 'resource' to 'resort'.

**Answer: correction included in the new text.**

Line 64: be consistent with how you write the 'beta' for beta-lactamases.

It would be more accurate to refer to the gene as blaNDM, and the allele as blaNDM-1 (so the blaNDM gene and its variants).

**Answer: in the new we homogenized the nomenclature of beta-lactamases.**

Line 68: I think this is the first mention of K. pneumoniae so Klebsiella should be written fully.

**Answer: correction made as suggested**

Line 70: the phrase 'causing problems in every hospital they have reached' is a bit over the top, and not something we can support with evidence.

**Answer: this phrase was eliminated in the new text**

Lines 77-78: you refer to transposons and then list some insertion sequences.

**Answer: correction made as suggested.**

Line 372-373: some italicising and bolding has over-run.

**Answer: correction made.**
